# Addition of Bioactive Glass Decreases Setting Time and Improves Antibacterial Properties of Mineral Trioxide Aggregate

**DOI:** 10.1155/2024/4190647

**Published:** 2024-07-23

**Authors:** Amin Salem Milani, Faezeh Hadinia, Yashar Rezaei, Mohammad Hossein Soroush Barhaghi, Kamal Attari, Ahmad Nouroloyouni

**Affiliations:** ^1^Endodontic Department, Faculty of Dentistry, Tabriz University of Medical Sciences, Tabriz, Iran; ^2^Department of Dental Biomaterials, Faculty of Dentistry, Tabriz University of Medical Sciences, Tabriz, Iran; ^3^Department of Medical Bacteriology & Virology, Faculty of Medicine, Tabriz University of Medical Sciences, Tabriz, Iran; ^4^Department of Orofacial Pain and Dysfunction, University of California, Los Angeles, CA, USA

## Abstract

**Objectives:**

This study aimed to assess the effect of addition of bioactive glass (BG) on the setting time and antibacterial activity of mineral trioxide aggregate (MTA) against Enterococcus faecalis (E. faecalis).

**Materials and Methods:**

In this in vitro study, BG was synthesized by the sol-gel technique and added to MTA powder in certain ratios. Three groups of specimens were fabricated from pure MTA, MTA mixed with 10wt% BG, and MTA mixed with 20wt% BG. The setting time of specimens was measured according to ISO9917-2007. Direct contact test was used to assess the antimicrobial activity of the three groups against E. faecalis. Data were analyzed by repeated measures ANOVA (alpha = 0.05).

**Results:**

Addition of BG (in both concentrations) to MTA decreased its setting time and improved its antibacterial activity against E. faecalis (*p* < 0.05). By an increase in concentration of BG (20%), the antimicrobial activity further improved (*p* < 0.05).

**Conclusion:**

Addition of BG to MTA in 10wt% and 20wt% concentrations decreased its setting time and improved its antibacterial activity against E. faecalis.

## 1. Introduction

Preliminary studies regarding bioactive glasses (BGs) were carried out in the University of Florida, which led to commercial production of BG 45S5 by Hench [1]. The BG 45S5 is composed of 45wt% SiO2, 24.5wt% Na2O, 24.4wt% CaO, and 6wt% P2O5. The aforementioned six oxides are the main constituents of contemporary BGs as well [1]. Bioactivity is defined as the capacity of a material to “elicit a specific biological response at the interface of the material which results in the formation of a bond between the tissues and the material” [2]. BGs can be synthesized by the same production methods used for silicate glasses. In these methods, the main oxides or compounds that produce oxides after degradation are mixed in certain ratios and melted at high temperatures to create a homogenous mixture. After cooling, glass is formed. The sol-gel technique, which is performed at room temperature, is a well-accepted method commonly used for synthesis of BGs [3].

BGs have unique properties that are ideal for medical purposes [4]. Thus, they are increasingly used as bioactive materials in bone tissue engineering, as a pulp capping agent [5–8], in pulpotomy [9–11], dental implant coating [12–14], and endodontic sealers [15, 16].

BGs can bond to both hard tissue (bone) and soft tissue. When exposed to biological fluids, BG forms a carbonated hydroxyapatite layer through intercellular reactions, that form a strong bond between BG and bone [13]. In addition to bonding to bone, BG has osteoinductive properties and enhances the adhesion, proliferation, and differentiation of osteoblasts. Moreover, it induces the differentiation of mesenchymal bone cells [17].

Evidence shows that BG can also induce angiogenesis both in vitro and in vivo [18].

Anti-inflammatory and antimicrobial properties are among other favorable properties of BG. Also, BG releases ions that induce osteogenesis, enhance the adhesion of bone cells, induce collagen synthesis, and increase the production of IGF-1 and IGF-2 by cells [19, 20].

Since BG has high compressive strength, it can be used to mechanically reinforce materials with low mechanical strength [21]. Considering the mechanism of bioactivity of BG, smaller size of glass particles increases the contact area and subsequently the bioactivity of BG. In other words, BGs with smaller particles have higher ionic release. Thus, nano-scale size of particles would significantly improve the properties of BG. The sol-gel technique synthesizes nano-scale BG [22]. Rezaei et al. [23] indicated that the bioactive glass which was synthesized in the study was able to elicit suitable biological response and showed improvement in bioactivity a few days after immersion in SBF solution.

As mentioned earlier, BG is highly biocompatible and induces osteogenesis through different mechanisms. Also, it has anti-inflammatory, antimicrobial, and favourable mechanical properties [3, 17, 19, 24]. Thus, aside from its application in fabrication of tissue engineering scaffolds [24], it may have the potential for use in apicoectomy. To date, several retrofill materials have been used for apicoectomy. Biologically, mineral trioxide aggregate (MTA) is superior to other materials for this purpose. However, it has difficult handling, delayed setting, risk of wash-out, and insignificant antibacterial activity [26]. Different methods have been used to improve the properties of MTA and address its drawbacks [27–30]. Mendoza Herrera et al. [31] evaluated the effect of addition of a type of BG to MTA and concluded that addition of BG decreased microleakage and improved the mechanical properties of MTA. Floresa-Ledesma et al. [32] evaluated the physical properties of MTA after addition of BG. In this study, addition of wollastonite and bioactive glass to a MTA-like cement caused reduction of compressive strength, setting time, and radiopacity. Solubility was decreased when wollastonite was added and it increased in bioactive glass group but it was less than 3% limit for all cement groups as defined by ISO 6876.

Floresa-Ledesma et al. [33] assessed effect of addition of bioactive materials on physical properties, marginal adaptation, and bioactivity Mineral Trioxide Aggregate-like cement.

They indicated that addition of wollastonite or bioactive glass to MTA decreased compressive strength and enhanced marginal adaptation but had no effect on solubility.

Kim et al. [34] analyzed effect of bioactive glass addition on the physical properties of Mineral Trioxide Aggregate and reported that compressive strength, setting time, and dentin push-out bond strength was improved when BG was added. The solubility was more in the BG/MTA groups comparison with control group.

Considering the gap of information on this topic, this study aimed to assess the effect of addition of BG on the setting time and antibacterial activity of MTA against Enterococcus faecalis (E. faecalis).

## 2. Materials and Methods

This experimental study was approved by the ethics committee of Tabriz University of Medical Sciences (TBZMED.VCR.REC.1399.416).

### 2.1. Synthesis of BG

The sol-gel technique was used for the synthesis of BG. For this purpose, 13.33 g of tetra-ethyl orthosilicate was added to 30 mL of 0.1 molar nitric acid; 30 minutes of time was allowed for acidic hydrolysis of tetra-ethyl orthosilicate. Next, 0.91 g of triethyl phosphate was added to the solution and stirred by a magnetic stirrer for 45 minutes to obtain a homogenous solution. Next, 4.96 g of calcium nitrate was added to the solution and allowed 45 minutes to completely dissolve; 1.28 g of magnesium nitrate and 1.05 g of strontium nitrate were also added separately to the solution and allowed 45 minutes to dissolve. The obtained mixture was stirred by 1 hour and stored in a Teflon container at room temperature for 1 week to allow completion of hydrolysis reactions. It was then heated at 70°C for 3 days for completion of the procedure. Next, water was eliminated, and a small hole was created in the container lid to allow leakage of gas. Gel was heated at 120°C for 2 days for complete elimination of water. Finally, the gel was heated at 700°C for 24 hours for stabilization and elimination of nitrate [28].

### 2.2. Specimen Preparation

Three groups of specimens were evaluated in this study. In group one, 20wt% BG powder was added to MTA (Salamifar Dental Supply, Tehran, Iran). In group two, 10wt% BG powder was added to MTA, and pure MTA was used in the third group. The mixture was placed in stainless-steel ring molds with 5 mm height immediately after mixing.

### 2.3. Measurement of Setting Time

The setting time of specimens was measured according to ISO 9917-1:2007 [35] 1 minute after mixing. A Vicat indenter (Humboldt Mfg Co, Schiller Park, IL) with 1 ± 0.1 mm diameter and 400 ± 5 g weight was used for this purpose. After 30 seconds, the indenter was vertically pressed on the surface of specimens for 5 seconds, and this process was repeated once every minute until the indenter no longer left an indentation on the specimen surface. The time interval between completion of mixing and the aforementioned time was recorded as the setting time. Testing was repeated in triplicate for each specimen, and the mean value was calculated and reported.

### 2.4. Assessment of Antibacterial Activity

E. faecalis (ATCC29212) was used for assessment of antibacterial activity by the direct contact test (DCT). A 96-well polystyrene plate (Becton Dickinson and company, Franklin Lakes, NJ, USA) was used for this purpose.

MTA powder mixed with 20wt% BG, MTA powder mixed with 10wt% BG, and pure MTA powder were mixed with sterile distilled water in 1 : 3 ratio under aseptic conditions and applied at the bottom of 8 wells (one row) of a 96-well plate in 2 mm thickness (coating). The plate was then incubated at 37°C and 100% humidity for 72 hours. Next, 10 µL of bacterial suspension with 0.5 McFarland standard concentration (containing 1-2 × 10^8^ colony forming units/mL) was added to the wells.

The first negative control group included 8 wells with the respective specimens at the bottom of the wells but without bacterial suspension.

The second negative control group included 8 wells without the specimens and without bacterial suspension.

The positive control group included 8 wells without the respective specimens but with bacterial suspension.

One hour after incubation at 37°C and drying of bacterial suspension in contact with the specimens, 245 *μ*L of sterile brain heart infusion (BHI) broth were added to each of the test and control wells, and vortexed for 2 minutes. Next, 15 *μ*L of the solution in each well was collected and transferred to another well in another plate, and 215 *μ*L of BHI was added and vortexed for 2 minutes.

To assess the bacterial proliferation, the plates were placed in a spectrophotometer (BioTek/Model 05404-0998/United States) with 630 nm wavelength. The optical density (OD) was read at 4 hours after vortexing, and once every hour after that for 3 hours. The plates were incubated at 37°C during the time interval between the readings. This test was also repeated in triplicate.

### 2.5. Statistical Analysis

Normal distribution of data was confirmed by the Kolmogorov–Smirnov test (*p* > 0.05). Thus, comparisons were made by repeated measures ANOVA. Data were analyzed using SPSS version 17 (SPSS Inc., IL, USA) at 0.05 level of significance.

## 3. Results

### 3.1. Setting Time

Table 1 presents the setting time of MTA in the three groups. As shown, by an increase in weight percentage of BG, the setting time of specimens decreased.

### 3.2. Antibacterial Activity

#### 3.2.1. Pure MTA Group

Table 2 presents the mean OD of pure MTA group in comparison with the control groups. Repeated measures ANOVA showed that the interaction effect of time and group on OD was significant (*p* < 0.001), such that in the first and second negative control groups, OD remained the same over time, but increased with time in other groups. The effect of time on OD was also significant (*p* < 0.001) such that the mean OD remained the same in the first and second negative control groups, but increased with time in other groups. A significant difference existed among the groups in OD (*p* < 0.001) such that the positive control group had the highest OD.

#### 3.2.2. MTA + 10% BG Group

Table 3 presents the mean OD of MTA + 10% BG group in comparison with the control groups. Repeated measures ANOVA showed that the interaction effect of time and group on OD was significant (*p* < 0.001), such that the mean OD remained constant over time in the first and second negative control groups but increased with time in other groups. The effect of time on OD was also significant (*p* < 0.001). OD increased with time except in the first and second negative control groups. A significant difference existed in OD of the groups (*p* < 0.001) such that the mean OD of the positive control group was the highest.

#### 3.2.3. MTA + 20% BG Group

Table 4 presents the mean OD of MTA + 20% BG group in comparison with the control groups. Repeated measures ANOVA showed that the interaction effect of time and group on OD was significant (*p* < 0.001), such that the mean OD remained constant over time in the first and second negative control groups but increased with time in other groups. The effect of time on OD was also significant (*p* < 0.001). OD increased with time except in the first and second negative control groups. A significant difference existed in OD of the groups (*p* < 0.001) such that the mean OD of the positive control group was the highest.

### 3.3. Comparison of the OD of the Three Study Groups

**Figure 1:**
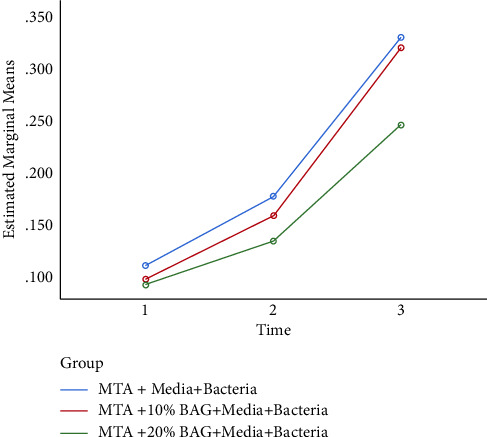
Optical density of the three groups over time.

Table 5 presents the mean OD of the three study groups at different time points. Repeated measures ANOVA showed that the interaction effect of time and group on OD was significant (*p*=0.001). The effect of time on OD was also significant (*p* < 0.001), such that OD increased with time in all three groups. A significant difference existed in OD of the study groups (*p*=0.008) such that the OD of pure MTA was the highest in all three readings and the OD of MTA + 20% BG was the lowest (Figure 1).

## 4. Discussion

This study assessed the effect of addition of BG on the setting time and antibacterial activity of MTA against E. faecalis. The results showed that addition of BG to MTA significantly decreased the setting time. Floresa-Ledesma et al. [32] evaluated the physical properties of MTA after addition of BG. They demonstrated that addition of BG decreased the setting time of MTA and 10wt% BG caused the greatest reduction in setting time (from 18 minutes to 20 seconds to 14 minutes) and also indicated that supplementation of BG more than 10wt% decreased the compressive strength of MTA without reducing the biological properties. Their results were similar to the present findings since 10wt% BG decreased the setting time of MTA from 8 minutes to 55 seconds to 6 minutes and 5 seconds. Addition of 20wt% BG further decreased the setting time to 5 minutes and 18 seconds. The decrease in setting time with the addition of BG possibly affects the alumino-silicate particles, as they are the first to react during the hydration process. It has been reported that particle shape and size of calcium silicates constituting MTA and Portland cements are essential for their properties. Smaller particles have more surface area and they thus present higher reactivity, and form calcium silicates hydrates and calcium hydroxide.

Kim et al. [34] showed that addition of 1%, 2%, 5%, and 10% BG to MTA decreased its setting time, and maximum reduction was observed in 1wt% group (42 minutes and 52 seconds).They reported that addition of 2 and/or 5wt% of BG improved physical properties and the most favorable BG concentration which delivers both mechanical and biological optimal results might be ranged from 2 to 5wt%.

Morgental et al. [36] indicated that set MTA had no antimicrobial effect on E.faecalis. The present study assessed the effect of addition of BG on antibacterial activity of MTA against E. faecalis. The results showed that addition of BG decreased the proliferation of E. faecalis and this reduction was greater in 20wt% BG compared with 10wt% BG. Over time, MTA +20wt% BG showed the highest antibacterial activity, while pure MTA showed the lowest antibacterial activity. The obtained results indicated that although pure MTA had antibacterial effect on E. faecalis, this effect was further enhanced by addition of BG in a concentration-dependent manner. Mehrvarzfar et al. [37] compared the antimicrobial effects of BG 45S5 and calcium hydroxide on E. faecalis, and found no significant difference in their antimicrobial activity at 1 hour and both materials showed improved antimicrobial activity with time (72 hours). They noted that particle size of Bioglass influences the antimicrobial activity and smaller particles provide broader exposed contact surface; therefore, more alkaline material is emancipated from the particle surface and increases the antimicrobial effect. In addition, presence of Ca, Si, and Na PO4 ions and cations in liquid medium and their emancipation from glass might lead to increase in pH and antimicrobial activity.

Mariyam et al. [38] reported that existence of Calcium Hydroxide and high alkaline pH of WMTA causes the antibacterial effect and OH^−^ ion could possibly be a cause of the cytoplasmic membrane and other cellular constituents break down, which leads to cell death but not enough to eradicate P. aeruginosa and E. faecalis.

Bioactive glass increases the pH level; therefore, addition of BG to Mineral Trioxide Aggregate will further increase the pH level. Difference between pH level of MTA and pH level of BG-added MTA needs further investigations. In the present study, DCT was used to evaluate the antimicrobial property. This method is a reliable antibacterial test that has many advantages over some commonly used antibacterial tests like the agar-diffusion test [39]. Other methods such as agar-diffusion test (ADT) have also been used with some major drawbacks. There may be chemical interactions between media and testing materials, and there is no study definitely correlating the inhibition zone diameters in ADT with clinical performance of disinfectants. Level of the pH has an important role in the antibacterial property of some sealers. Zone of inhibition diameter in agar-diffusion test (ADT) is affected by the buffering capacity of agar [39].

Bolhari et al. [40] evaluated the effect of addition of 10% and 15% fluorohydroxyapatite on the antibacterial activity of MTA against E. faecalis in vitro by the agar disc diffusion test, biofilm inhibitory assay, and DCT. The DCT revealed that after 24 hours, a growth inhibition zone was noted around MTA, MTA + 10% fluorohydroxyapatite, and MTA + 15% fluorohydroxyapatite, indicating their antimicrobial activity. Also, the colony count significantly decreased at 24 and 72 hours. MTA + 15% fluorohydroxyapatite had the highest antimicrobial activity at 72 hours. The antimicrobial activity of MTA + fluorohydroxyapatite increased with time.

## 5. Conclusion

The present results indicated that addition of 10wt% and 20wt% BG to MTA decreased its setting time and increased its antibacterial activity against E. faecalis. The antibacterial activity further improved by an increase in concentration of BG from 10wt% to 20wt%. Further research is recommended to evaluate the antibacterial activity against other bacterial species and also to investigate accurate mechanism of antibacterial effect of BG added Mineral Trioxide Aggregate [25].

## Figures and Tables

**Table 1:** Setting time (in minutes) of MTA in the three groups.

Group	Order of test	Setting time	Mean setting time
MTA	First test	9: 24	8: 55
	Second test	9: 47	
	Third test	7: 33	

MTA + 10% BG	First test	6: 51	6: 05
	Second test	5: 46	
	Third test	5: 40	

MTA + 20% BG	First test	5: 38	5: 18
	Second test	5: 36	
	Third test	4: 40	

**Table 2:** Mean OD of pure MTA group in comparison with the control groups (*n* = 24).

Time (H)	Group	Mean	Std. Deviation	Time ^*∗*^group	Time	Group
4	MTA	0.111	0.028	<0.001 ^*∗*^	<0.001 ^*∗*^	<0.001 ^*∗*^
	Positive control	0.118	0.015			
	First negative control	0.078	0.002			
	Second negative control	0.078	0.004			
5	MTA	0.177	0.059			
	Positive control	0.217	0.048			
	First negative control	0.078	0.002			
	Second negative control	0.078	0.003			
6	MTA	0.330	0.099			
	Positive control	0.393	0.060			
	First negative control	0.078	0.002			
	Second negative control	0.078	0.003			

^*∗*^Repeated measures ANOVA.

**Table 3:** Mean OD of MTA + 10% BG group in comparison with the control groups (*n* = 24).

Time (H)	Group	Mean	Std. Deviation	Time ^*∗*^group	Time	Group
4	MTA + 10% BG	0.098	0.008	<0.001 ^*∗*^	<0.001 ^*∗*^	<0.001 ^*∗*^
	Positive control	0.118	0.015			
	First negative control	0.076	0.002			
	Second negative control	0.078	0.003			
5	MTA + 10% BG	0.159	0.030			
	Positive control	0.217	0.048			
	First negative control	0.076	0.003			
	Second negative control	0.078	0.003			
6	MTA + 10% BG	0.319	0.056			
	Positive control	0.393	0.060			
	First negative control	0.076	0.004			
	Second negative control	0.078	0.004			

^*∗*^Repeated measures ANOVA.

**Table 4:** Mean OD of MTA + 20% BG group in comparison with the control groups (*n* = 24).

Time (H)	Group	Mean	Std. Deviation	Time ^*∗*^group	Time	Group
4	MTA + 20% BG	0.09	0.02	<0.001 ^*∗*^	<0.001 ^*∗*^	<0.001 ^*∗*^
	Positive control	0.12	0.02			
	First negative control	0.08	0.00			
	Second negative control	0.08	0.00			
5	MTA + 20% BG	0.13	0.05			
	Positive control	0.22	0.05			
	First negative control	0.08	0.00			
	Second negative control	0.08	0.00			
6	MTA + 20% BG	0.25	0.13			
	Positive control	0.39	0.06			
	First negative control	0.08	0.01			
	Second negative control	0.08	0.00			

^*∗*^Repeated measures ANOVA.

**Table 5:** Mean OD of the three study groups at different time points (*n* = 24).

Time (H)	Group	Mean	Std. Deviation	Time ^*∗*^group	Time	Group
4	MTA	0.111	0.028	0.001 ^*∗*^	<0.001 ^*∗*^	0.008 ^*∗*^
	MTA + 10% BG	0.098	0.008			
	MTA + 20% BG	0.093	0.016			
5	MTA	0.177	0.059			
	MTA + 10% BG	0.159	0.030			
	MTA + 20% BG	0.134	0.054			
6	MTA	0.330	0.099			
	MTA + 10% BG	0.319	0.056			
	MTA + 20% BG	0.245	0.126			

^*∗*^Repeated measures ANOVA.

## Data Availability

The data that support the findings of this study are available from the corresponding author upon reasonable request.

## References

[1] Hench L. L. (2006). The story of Bioglass. *Journal of Materials Science: Materials in Medicine*.

[2] Corral Nuñez C., Covarrubias C., Fernandez E., Oliveira O. B. (2017). Enhanced bioactive properties of Biodentine TM modified with bioactive glass nanoparticles. *Journal of Applied Oral Science*.

[3] Jones J. R. (2013). Review of bioactive glass: from Hench to hybrids. *Acta Biomaterialia*.

[4] Baino F., Fiorilli S., Vitale-Brovarone C. (2016). Bioactive glass-based materials with hierarchical porosity for medical applications: review of recent advances. *Acta Biomaterialia*.

[5] Oguntebi B., Clark A., Wilson J. (1993). Pulp capping with Bioglass® and autologous demineralized dentin in miniature swine. *Journal of Dental Research*.

[6] Haghgoo R., Naderi N. J. (2007). Comparison of calcium hydroxide and bioactive glass after direct pulp capping in primary teeth. *Journal of Dentistry of Tehran University of Medical Sciences*.

[7] Goldberg M., Six N., Decup F. (2001). Application of bioactive molecules in pulp-capping situations. *Advances in Dental Research*.

[8] Long Y., Liu S., Zhu L., Liang Q., Chen X., Dong Y. (2017). Evaluation of pulp response to novel bioactive glass pulp capping materials. *Journal of Endodontics*.

[9] Salako N., Joseph B., Ritwik P., Salonen J., John P., Junaid T. A. (2003). Comparison of bioactive glass, mineral trioxide aggregate, ferric sulfate, and formocresol as pulpotomy agents in rat molar. *Dental Traumatology*.

[10] Jabarifar S. E., Khademi A., Ghasemi D. (2004). Success rate of formocresol pulpotomy versus mineral trioxide aggregate in human primary molar tooth. *Journal of Research in Medical Sciences*.

[11] Haghgoo R., Ahmadvand M. (2016). Evaluation of pulpal response of deciduous teeth after direct pulp capping with bioactive glass and mineral trioxide aggregate. *Contemporary Clinical Dentistry*.

[12] Covarrubias C., Mattmann M., Von Marttens A. (2016). Osseointegration properties of titanium dental implants modified with a nanostructured coating based on ordered porous silica and bioactive glass nanoparticles. *Applied Surface Science*.

[13] Mistry S., Roy R., Kundu B. (2016). Clinical outcome of hydroxyapatite coated, bioactive glass coated, and machined Ti6Al4V threaded dental implant in human jaws: a short-term comparative study. *Implant Dentistry*.

[14] Xuereb M., Camilleri J., Attard N. J. (2015). Systematic review of current dental implant coating materials and novel coating techniques. *International Journal of Prosthodontics*.

[15] Tanomaru-Filho M., Torres F. F., Chávez-Andrade G. M. (2017). Physicochemical properties and volumetric change of silicone/bioactive glass and calcium silicate–based endodontic sealers. *Journal of Endodontics*.

[16] Heid S., Stoessel P. R., Tauböck T. T., Stark W. J., Zehnder M., Mohn D. (2016). Incorporation of particulate bioactive glasses into a dental root canal sealer. *Biomedical glasses*.

[17] Tavakolizadeh A., Ahmadian M., Fathi M. H., Doostmohammadi A., Seyedjafari E., Ardeshirylajimi A. (2017). Investigation of osteoinductive effects of different compositions of bioactive glass nanoparticles for bone tissue engineering. *ASAIO Journal*.

[18] Kargozar S., Baino F., Hamzehlou S., Hill R. G., Mozafari M. (2018). Bioactive glasses: sprouting angiogenesis in tissue engineering. *Trends in Biotechnology*.

[19] Zhang D., Leppäranta O., Munukka E. (2010). Antibacterial effects and dissolution behavior of six bioactive glasses. *Journal of Biomedical Materials Research Part A*.

[20] Xynos I. D., Edgar A. J., Buttery L. D., Hench L. L., Polak J. M. (2000). Ionic products of bioactive glass dissolution increase proliferation of human osteoblasts and induce insulin-like growth factor II mRNA expression and protein synthesis. *Biochemical and Biophysical Research Communications*.

[21] Kokubo T. (1991). Bioactive glass ceramics: properties and applications. *Biomaterials*.

[22] Li R., Clark A., Hench L. (1991). An investigation of bioactive glass powders by sol‐gel processing. *Journal of Applied Biomaterials*.

[23] Rezaei Y., Moztarzadeh F., Shahabi S., Tahriri M. (2014). Synthesis, characterization, and in vitro bioactivity of sol-gel-derived SiO2–CaO–P2O5–MgO-SrO bioactive glass. *Materials Science and Engineering: C*.

[24] Höland W. (1997). Biocompatible and bioactive glass-ceramics—state of the art and new directions. *Journal of Non-crystalline Solids*.

[25] Rahaman M. N., Day D. E., Bal B. S., Fu Q., Jung S. B., Bonewald L. F. (2011). Bioactive glass in tissue engineering. *Acta Biomaterialia*.

[26] Parirokh M., Torabinejad M. (2010). Mineral trioxide aggregate: a comprehensive literature review--Part I: chemical, physical, and antibacterial properties. *Journal of Endodontics*.

[27] Salem Milani A., Radmand F., Rahbani B., Hadilou M., Haji Abbas Oghli F., Salehnia F. (2023). Effect of different mixing methods on physicochemical properties of mineral trioxide aggregate: a systematic review. *Int J Dent*.

[28] Rahimi S., Salarinasab S., Ghasemi N., Rahbarghazi R., Shahi S., Salem Milani A. (2019). In vitro induction of odontogenic activity of human dental pulp stem cells by white Portland cement enriched with zirconium oxide and zinc oxide components. *Journal of Dental Research, Dental Clinics, Dental Prospects*.

[29] Eskandarinezhad M., Shahveghar-Asl N., Sharghi R. (2017). Sealing efficacy of mineral trioxide aggregate with and without nanosilver for root end filling: a bacterial leakage study. *J Clin Exp Dent*.

[30] Salem M. A., Froughreyhani M., Charchi Aghdam S., Pournaghiazar F., Asghari J. M. (2013). Mixing with propylene glycol enhances the bond strength of mineral trioxide aggregate to dentin. *Journal of Endodontics*.

[31] Mendoza Herrera M. C., Flores-Ledesma A., Barceló Santana F. H. (2018). Microleakage in MTA-type dental cement modified with wollastonite and bioactive glass. *Revista Odontológica Mexicana*.

[32] Flores-Ledesma A., Santana F. B., Bucio L., Arenas-Alatorre J. A., Faraji M., Wintergerst A. M. (2017). Bioactive materials improve some physical properties of a MTA-like cement. *Materials Science & Engineering, C: Materials for Biological Applications*.

[33] Flores-Ledesma A., Tejeda-Cruz A., Moyaho-Bernal M. A. (2023). Physical properties, marginal adaptation and bioactivity of an experimental mineral trioxide aggregate-like cement modified with bioactive materials. *Journal of Oral Science*.

[34] Kim J., Kim H. J., Chang S. W. (2021). Effect of bioactive glass addition on the physical properties of mineral trioxide aggregate. *Biomaterials Research*.

[35] International Standards Organization *ISO 9917-1: 2007: Water-Based Cements-Part 1: Powder/liquid Acid-Base Cements*.

[36] Morgental R. D., Vier‐Pelisser F. V., Oliveira S. Dd, Antunes F. C., Cogo D. M., Kopper P. (2011). Antibacterial activity of two MTA‐based root canal sealers. *International Endodontic Journal*.

[37] Mehrvarzfar P., Akhavan H., Rastgarian H., Akhlagi N. M., Soleymanpour R., Ahmadi A. (2011). An in vitro comparative study on the antimicrobial effects of bioglass 45S5 vs. calcium hydroxide on *Enterococcus faecalis*. *Iranian Endodontic Journal*.

[38] Mariyam M., Sunarintyas S., Nuryono N. (2023). Improving mechanical, biological, and adhesive properties of synthesized mineral trioxide aggregate by adding chitosan. *Inorganic Chemistry Communications*.

[39] Milani A. S., Moeinian A., Barhaghi M. H., Abdollahi A. A. (2020). Evaluation of the film thickness and antibacterial property of mineral trioxide aggregate mixed with propylene glycol as a root canal sealer. *Dental Research Journal*.

[40] Bolhari B., Sooratgar A., Pourhajibagher M., Chitsaz N., Hamraz I. (2021). Evaluation of the antimicrobial effect of mineral trioxide aggregate mixed with fluorohydroxyapatite against *E. faecalis* in vitro. *The Scientific World Journal*.

